# Can information from citizen science data be used to predict biodiversity in stormwater ponds?

**DOI:** 10.1038/s41598-020-66306-0

**Published:** 2020-06-10

**Authors:** Frank Johansson, Jani Heino, Paul Coiffard, Richard Svanbäck, Jacob Wester, Luis Mauricio Bini

**Affiliations:** 1 Department of Ecology and Genetics, Animal Ecology, Uppsala University, Norbyvägen 18D, 752 36 Uppsala, Sweden; 2 Finnish Environment Institute, Freshwater Centre, Paavo Havaksen Tie 3, FI-90570 Oulu, Finland; 3 Departamento de Ecologia, Universidade Federal de Goiás, Goiânia, 74690-900 GO Brazil

**Keywords:** Biodiversity, Community ecology, Conservation biology, Freshwater ecology, Urban ecology, Wetlands ecology

## Abstract

Citizen science data (CSD) have the potential to be a powerful scientific approach to assess, monitor and predict biodiversity. Here, we ask whether CSD could be used to predict biodiversity of recently constructed man-made habitats. Biodiversity data on adult dragonfly abundance from all kinds of aquatic habitats collected by citizen scientists (volunteers) were retrieved from the Swedish Species Observation System and were compared with dragonfly abundance in man-made stormwater ponds. The abundance data of dragonflies in the stormwater ponds were collected with a scientific, standardized design. Our results showed that the citizen science datasets differed significantly from datasets collected scientifically in stormwater ponds. Hence, we could not predict biodiversity in stormwater ponds from the data collected by citizen scientists. Using CSD from past versus recent years or from small versus large areas surrounding the stormwater ponds did not change the outcome of our tests. However, we found that biodiversity patterns obtained with CSD were similar to those from stormwater ponds when we restricted our analyses to rare species. We also found a higher beta diversity for the CSD compared to the stormwater dataset. Our results suggest that if CSD are to be used for estimating or predicting biodiversity, we need to develop methods that take into account or correct for the under-reporting of common species in CSD.

## Introduction

In the face of increasing human pressure on biodiversity^1^, scientists need additional approaches to respond to the demand for information to guide environmental management, conservation planning and policymaking^2^. One such approach consists of the involvement of citizens (volunteers) to gather data for scientific purposes (see reviews in Dickinson *et al*.^3,4^). Citizen science data (CSD) are gathered by non-professionals, including biodiversity data across space and time^5^. Because many non-professional naturalists are involved in biodiversity data collection, one of the biggest advantages is that this approach generates a large amount of data^3^. These datasets have been successfully used in many studies, for example, in monitoring population trends of species, assessing the impacts of global warming on biodiversity, and forecasting species responses to forest management^6,7^. Hence, CSD are important in providing large amounts of data for scientists with little financial resources devoted to large-scale biodiversity surveys.

However, CSD may also have several drawbacks, mostly associated with the lack of a proper sampling design and accuracy of records^8^. Snäll *et al*.^9^ listed several of these drawbacks, such as: (1) population records only include presence, but not absence, (2) sampling effort varies over space and time, (3) spatial coverage might vary, (4) methods of collection might vary, (5) records of rare and common species might be biased towards rare or common species, and (6) detectability of species varies among volunteers. Because of these drawbacks, more studies are needed to examine how well CSD can be used to predict, for example, colonization of new habitats. Such new habitats could be created by environmental disturbance or deliberately by humans.

One type of new habitats created by humans are stormwater ponds. These ponds are water bodies constructed for capturing and storing water from rainstorms; they can be temporary or permanent. In general, stormwater ponds have a high biodiversity, especially when considering invertebrates and amphibians^10–14^. Because many stormwater ponds were recently constructed, and many more are being constructed^14,15^, it is interesting to ask whether knowledge of the distribution and abundance of organisms in the surrounding areas can be used to predict biodiversity in the ponds. Here, we investigate whether CSD from records of aquatic insects from the Swedish Species Observation System (www.artportalen.se) can be used to predict the biodiversity of insects in recently constructed stormwater ponds. We used adult dragonflies (Odonata: Zygoptera and Anisoptera) as our focal group of insect biodiversity. Dragonflies are intermediate consumers both in the aquatic and the terrestrial life stage, i.e., they are predators of smaller invertebrate prey and are preyed upon by larger invertebrate and vertebrate predators^16^. Because they have an intermediate position in the food web, they should represent overall patterns in aquatic biodiversity relatively well^16^. In addition, Odonata species richness is positively correlated with species richness of many, but not all, invertebrate taxa and vegetation abundance^17,18^.

To examine whether biodiversity patterns in stormwater ponds could be predicted from CSD, we used three approaches. First, we examined the similarity between biodiversity datasets (i.e., between CSD and those obtained in urban stormwater ponds) from past to recent years. Because Odonata have a short generation time (usually 1–2 years) and because they are good dispersers^16^, we expected that community patterns generated with data from recent years would be better predictors of community structure in stormwater ponds. Second, we examined how the inclusion of CSD covering different areas around the stormwater ponds predicted the Odonata diversity in the ponds. We expected that citizen science datasets covering an area with a larger diameter around our study area would be a better predictor since a large area covers more habitats. We also considered an alternative hypothesis, where predictability decreased with area, due to the distance decay of similarity in ecological communities, see e.g. Nekola & White^19^. Third, we examined whether including common or rare species affected the predictions. We expected that community patterns based on data from rare species would be more similar to the patterns based on our dataset (stormwater ponds) because these species are more actively sought by citizen scientists than common species^3,20,21^.

## Methods

### Stormwater pond data

**Figure 1:**
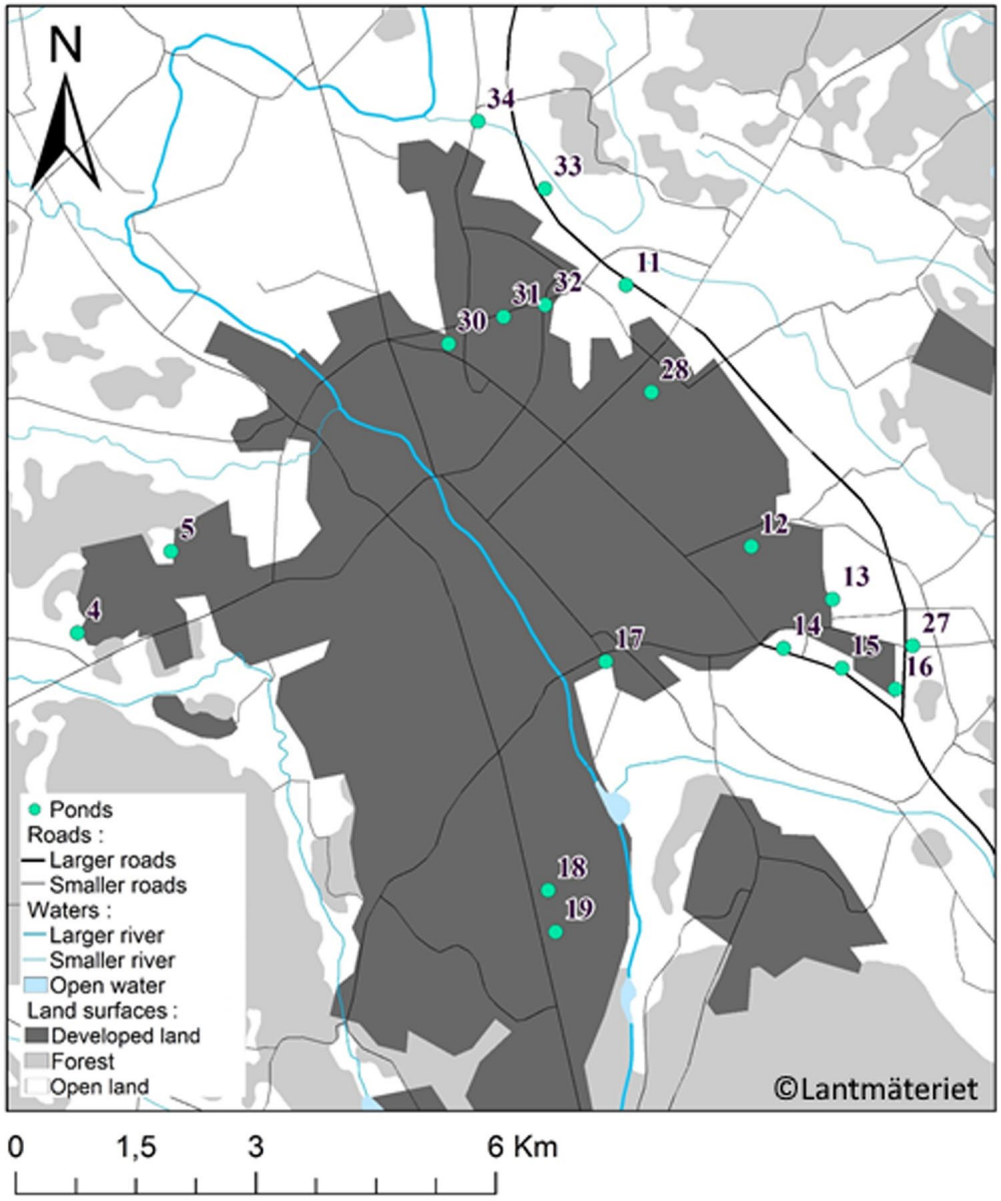
Map of the City of Uppsala, Sweden showing the location of the 18 stormwater ponds. The map was created in the software ArcGIS 10.0, ESRI Inc., Redlands CA (https://desktop.arcgis.com/en/) with permission from Lantmäteriet^43^ through Uppsala University (license number: I2018/00145).

Biodiversity data of dragonflies in 18 stormwater ponds were obtained in the city of Uppsala, Sweden in 2018. These man-made ponds were recently constructed (i.e. between 2004 and 2014). The city of Uppsala has 150 000 inhabitants and covers an area of 26 km², and all stormwater ponds that are filled with water all year round were used for this study (Fig. 1; for more details on methods and pond description, see Johansson *et al*.^22^). Dragonfly abundance was recorded every second week over a 10-week period by two trained researchers (P.C and J.W.), who walked one lap slowly around the ponds from 29 May until 5 August 2018. This period covers the emergence period of all species found at this latitude^23^. Most species were identified visually. However, for some species, identifications were made after capture by a butterfly net. The speed of walking was adjusted with respect to vegetation of the pond and by the abundance of dragonflies, such that the speed was slow at ponds with a lot of vegetation and a high abundance of dragonflies. Total numbers of adults (including mating pairs and ovipositing individuals) were counted and used for the subsequent analyses. No counts were done during cloudy, windy (>30 km/h) or rainy days and, therefore, the biweekly counts were shifted 1–2 days on two of the sampling occasions. For the analyses, the week with the highest number of individuals was used for each species. These ponds and a modified data set was used in a recent study by Johansson *et al*.^22^ and therefore pond description and some of the methods overlap slightly with the information given in that study^22^.

### Citizen science data (CSD)

We used the Swedish Species Observation System to extract records of dragonfly abundance based on CSD. These observations were collected by citizens in a non-standardized way, meaning that data were gathered opportunistically without standardized methods and controlling for sampling effort^24^. These observations included all water bodies in the study area, and thus CSD habitats might represent freshwater systems with a wider range of habitat characteristics. Unfortunately, the CSD did not have enough replicates of stormwater ponds, which would allow to control for other confounding factors (e.g., type of water body). However, we included only species that were recorded in our stormwater pond survey. Hence, species that did not occur in the stormwater ponds were excluded from the CSD set. When the number of individuals was available in the database, we used this number as our estimate, and when the record of a species only mentioned “observed”, we gave this record an abundance of 1. Two hundred forty-eight (248) localities were surveyed by citizens during 8 years. Furthermore, two species, *Calopteryx virgo* and *C. splendens*, are predominantly lotic specialists and typically do not occur in standing waters, such as stormwater ponds. They were therefore removed from the analyses. These species occurred in low numbers at the stormwater ponds (a total of 5 and 2 individuals for *C. virgo* and *C. splendens*, respectively).

**Figure 2:**
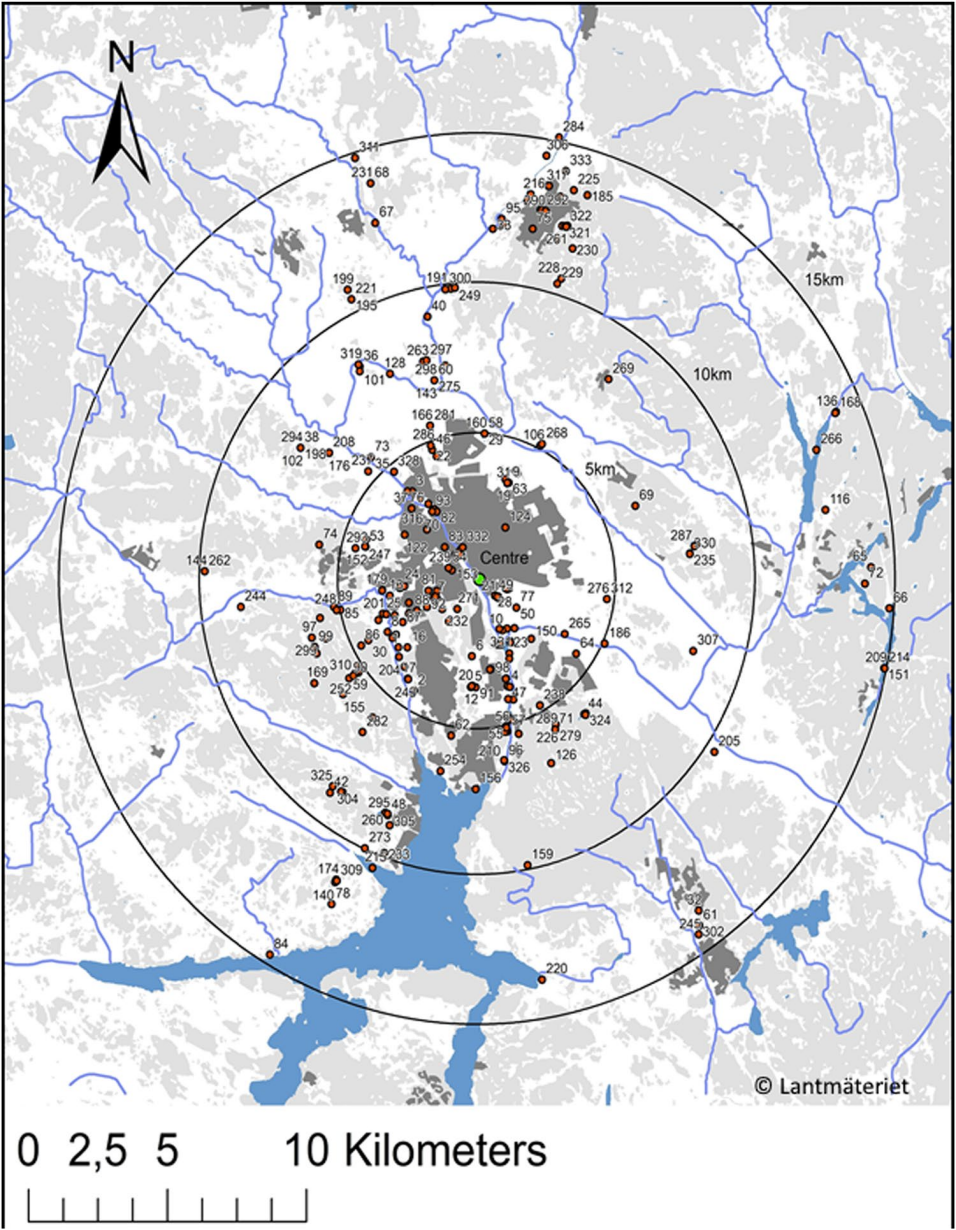
Distribution of citizen science data records (red dots). The green dot is the center of the study area and the circles show a 10, 20 and 30 km diameter from the center. Note that the dots for the 20 and 30 km only include the year 2017, while the 10 km radius include dots from 2010–2017. The map was created in the software ArcGIS 10.0, ESRI Inc., Redlands CA (https://desktop.arcgis.com/en/) with permission from Lantmäteriet^43^ through Uppsala University (license number: I2018/00145).

To evaluate whether CSD could be used to predict pond biodiversity, we extracted data covering different diameters around the center of the stormwater ponds in the city (Fig. 2). The center of the study area (59°50′51.3″N; 17°39′3.4″E) was estimated approximately as the centroid of the coordinates of the stormwater ponds. Data records from the database were extracted on a yearly basis from 2010 until 2017 to examine whether more recent CSD performed better in predicting biodiversity in the stormwater ponds. In addition, we also examined whether citizen data records covering a larger area around the city center improved the prediction of stormwater pond biodiversity. We did this by extracting and comparing data covering a diameter of 10, 20 or 30 km around the center of the stormwater ponds in the city (Fig. 2). This analysis was performed for the year 2017.

### Data analyses

We used a Canonical Analysis of Principal Coordinates (CAP; Anderson & Willis^25^) to investigate the differences in community structure between the stormwater pond (sampled by us in 2018) and the citizen datasets in terms of dragonfly species abundance and composition. CAP is a constrained ordination method and, therefore, it uses an a priori hypothesis to produce an ordination plot. This hypothesis can then be tested using a generalized discriminant analysis based on distances^26^. Our a priori hypothesis was represented by a categorical (explanatory) variable with levels representing the type of data (stormwater in 2018 and citizen datasets). CAP was based on the Bray-Curtis dissimilarity index on dragonfly abundance data. The resulting dissimilarity matrix was also used in a distance-based test for homogeneity of multivariate dispersions (PERMDISP; see Anderson,^27^; Anderson *et al*.^28^). This was done to test whether beta diversity values differed according to the type of the data. Since our main interest was focused on comparing the data obtained in the 2018 stormwater ponds with the data obtained by citizens, we carried out a set of planned comparisons. First, we repeated CAP to compare the 2018 dataset to each year separately (from 2010 to 2017) considering sites (surveyed by citizens) that were within a diameter of 10 km around the center of the stormwater ponds in the city. Second, we used CAP to compare the 2018 dataset with sites within diameters of 10 km, 20 km and 30 km around the center of the stormwater ponds. This analysis was restricted to the year 2017. Third, to evaluate the likely effect of a biased search for rarer species in the CSD, we also divided the dataset into two parts. The first part included the 16 most abundant species in our stormwater dataset (see Fig. S1), whereas the second part included the 11 rarer species. This splitting of the dataset was based on the location of an inflection point exhibited in a Whittaker plot (rank-abundance curve) and on an attempt to balance the number of rare and common species in the analyses. Thereafter, we ran independent analyses using these datasets. All analyses were carried out using functions (*vegdist*, *capscale* and *betadisper*) available in the vegan package^29^. Significance tests were based on 999 permutations.

## Results

Twenty-nine species of Odonata were found in the 18 studied stormwater ponds (Table 1), but only 27 were included in the main analyses since the two lotic *Calopteryx* species were excluded. The total number of species represents 61% of the Odonata species recorded in the province of Uppland in Sweden. The average species richness in the stormwater ponds was 10, with a range between 3 to 19. The most common species were *Libellula quadrimaculata*, *Sympetrum vulgatum*, *Lestes sponsa* and *Coenagrion puella*. Five species were only recorded in one pond (*Platycnemis pennipes*, *Aeshna mixta*, *Orthetrum coerulescens*, *Leucorrhinia pectoralis* and *Leucorrhinia rubicunda*; Table 1). Considering the CSD-set, the most common species were

**Table 1:** List of dragonfly species recorded by citizen scientists (from 2010 up to 2017) and in 18 stormwater ponds in the city of Uppsala, Central Sweden (2018). Also shown is the frequency of occurrence of each species (number of occurrences/number of sites). Note that the lotic species, *Calopteryx splendens* and *C. virgo*, were excluded from the statistical analyses.

Species/Years	2010	2011	2012	2013	2014	2015	2016	2017	2018
*Aeshna cyanea*	25.0	15.0	30.8	15.2	21.4	11.1	7.7	17.6	11.1
*Aeshna grandis*	16.7	25.0	46.2	27.3	7.1	0.0	15.4	17.6	61.1
*Aeshna juncea*	8.3	15.0	15.4	12.1	7.1	0.0	7.7	0.0	11.1
*Aeshna mixta*	0.0	0.0	7.7	12.1	0.0	0.0	3.8	0.0	5.6
*Aeshna serrata*	0.0	0.0	7.7	6.1	0.0	0.0	3.8	0.0	22.2
*Brachytron pratense*	0.0	0.0	0.0	6.1	7.1	0.0	0.0	11.8	22.2
*Cordulia aenea*	0.0	5.0	7.7	6.1	0.0	3.7	3.8	5.9	16.7
*Coenagrion hastulatum*	0.0	10.0	30.8	18.2	14.3	11.1	26.9	29.4	61.1
*Coenagrion lunulatum*	0.0	0.0	0.0	0.0	0.0	0.0	15.4	0.0	16.7
*Coenagrion puella*	0.0	0.0	23.1	9.1	21.4	14.8	23.1	11.8	77.8
*Coenagrion pulchellum*	8.3	5.0	23.1	18.2	14.3	22.2	19.2	17.6	61.1
*Calopteryx splendens*	8.3	15.0	23.1	12.1	14.3	11.1	23.1	23.5	11.1
*Calopteryx virgo*	8.3	25.0	7.7	45.5	21.4	29.6	19.2	17.6	27.8
*Enallagma cyathigerum*	0.0	5.0	15.4	9.1	7.1	7.4	23.1	17.6	66.7
*Erythromma najas*	8.3	5.0	23.1	9.1	7.1	7.4	19.2	5.9	27.8
*Ischnura elegans*	0.0	0.0	7.7	3.0	14.3	7.4	7.7	11.8	50.0
*Libellula depressa*	0.0	10.0	7.7	0.0	14.3	3.7	7.7	11.8	33.3
*Leucorrhinia pectoralis*	0.0	0.0	7.7	3.0	0.0	0.0	3.8	0.0	5.6
*Libellula quadrimaculata*	16.7	10.0	23.1	9.1	0.0	3.7	7.7	0.0	94.4
*Leucorrhinia rubicunda*	8.3	0.0	0.0	6.1	0.0	0.0	3.8	0.0	5.6
*Lestes sponsa*	0.0	15.0	15.4	24.2	14.3	11.1	11.5	11.8	83.3
*Orthetrum coerulescens*	0.0	0.0	0.0	0.0	0.0	0.0	0.0	0.0	5.6
*Platycnemis pennipes*	0.0	5.0	23.1	9.1	7.1	22.2	15.4	17.6	5.6
*Sympetrum danae*	0.0	5.0	23.1	12.1	7.1	11.1	11.5	11.8	16.7
*Sympetrum flaveolum*	0.0	5.0	23.1	6.1	7.1	3.7	3.8	0.0	38.9
*Sympecma fusca*	8.3	5.0	15.4	3.0	7.1	7.4	19.2	23.5	16.7
*Somatochlora metallica*	0.0	25.0	46.2	18.2	7.1	7.4	0.0	17.6	22.2
*Sympetrum sanguineum*	16.7	20.0	30.8	21.2	21.4	14.8	7.7	17.6	44.4
*Sympetrum vulgatum*	0.0	30.0	38.5	33.3	28.6	11.1	7.7	23.5	94.4

**Figure 3:**
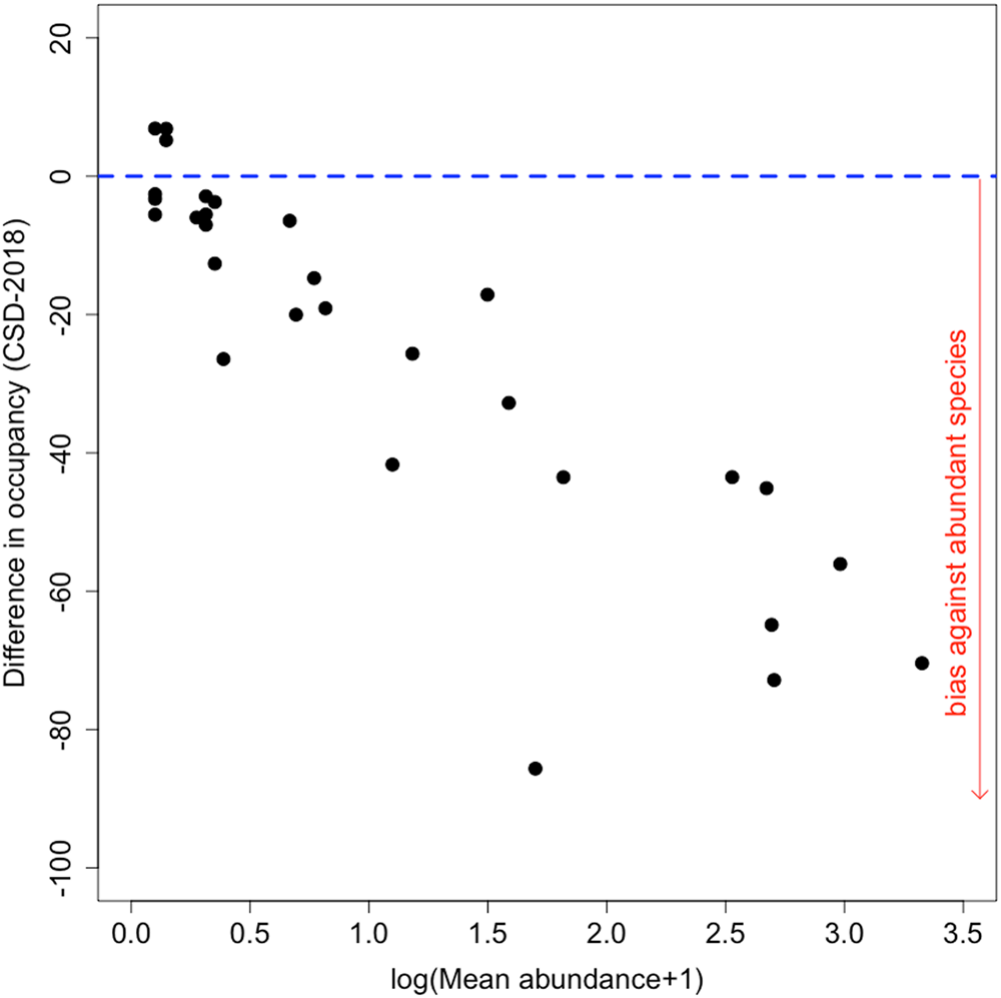
Relationship between the difference in occupancy given by the datasets [average of the species frequencies of occurrence over the years in the CSD - frequency of occurrence in the stormwater ponds (2018)] and the mean abundance in the stormwater ponds. Each point represents a species. The dashed line represents no difference and negative values indicate that occupancies as given in CSD were lower than those in the stormwater ponds.

*Aeshna cyanea* (2010), *Sympetrum vulgatum* (2011 and 2014), *Aeshna grandis* (2012), *Somatochlora metallica* (2012), *Calopteryx virgo* (2013 and 2015), *Coenagrion hastulatum* (2016 and 2017; Table 1). We found a strong negative relationship between the difference in occupancy given by the datasets (average of the species frequencies of occurrence over the years in the CSD - frequency of occurrence in the stormwater ponds) and the mean abundance in the stormwater ponds (Fig. 3; *r* = −0.88; *P* < 0.0001 with all species and *r* = −0.87; *P* < 0.0001 after removing *C. virgo* and *C. splendens*). This result indicates a bias against abundant species in the CSD.

**Figure 4:**
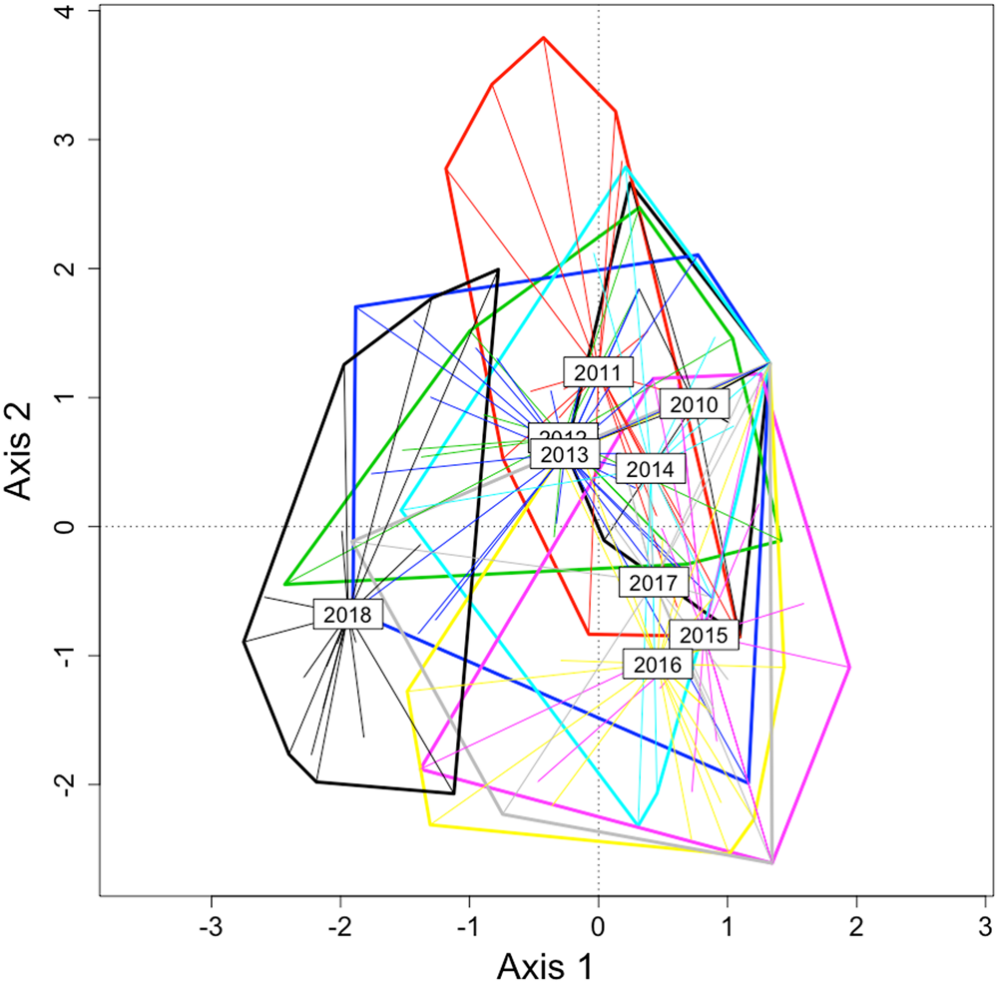
Ordination of sites (Canonical Analysis of Principal Coordinates) based on dragonfly species recorded in stormwater ponds (2018) and by citizen scientists (from 2010 up to 2017).

**Figure 5:**
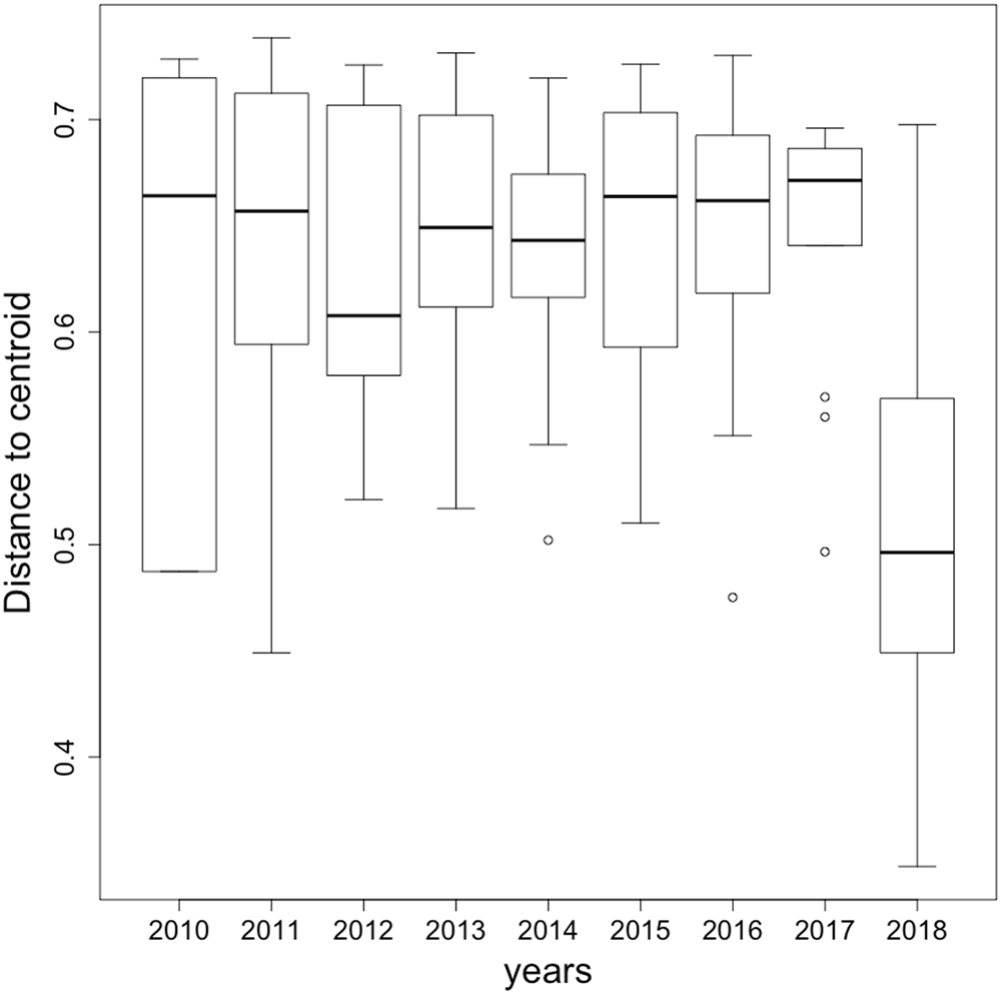
Distance to centroid within each year for citizen datasets (2010 to 2017) and for the stormwater ponds dataset (2018).

We found a significant difference between the citizen dataset (considering different years, 2010–2017) and the stormwater pond dataset from 2018 (Fig. 4; *F* = 1.97; *P* < 0.001), and the community structure from the more recent years of CSD-set were not more similar to that of the stormwater ponds community structure (Fig. 4). In addition, variations in community structure, i.e. beta diversity, as given by the citizen science datasets were much higher than that given by the 2018 stormwater pond dataset (Fig. 5; *F* = 6.77; *P* < 0.001). We also found that the differences among the datasets remained independently of the diameter (10, 20 and 30 km) used to extract the CSD (*F* = 2.61; *P* = 0.001). Running these analyses after including data on lotic species did not change the results qualitatively (results not shown).

**Figure 6:**
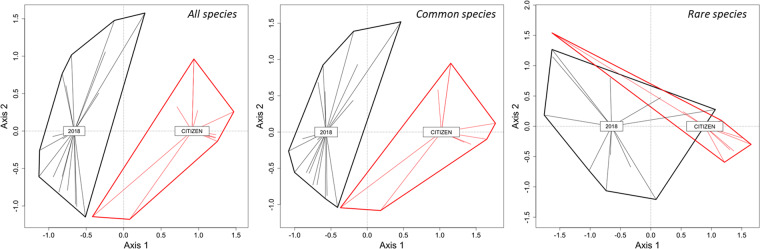
Ordination of sites (Canonical Analysis of Principal Coordinates) based on dragonfly species recorded in stormwater ponds (2018) and by citizen scientists (CITIZEN). Shown are the results obtained with all species (left), common species (center) and rare species (right) for the year 2017 (as an example).

After splitting the dataset according to species abundance (see Fig. S1), we found that the differences between citizen science datasets and the 2018 dataset were remained only when common species were considered in the analyses (Table 2; Fig. 6). In contrast, we did not find any significant difference in community structure (between CSD and 2018 dataset) when rare species were considered (Table 2; Fig. 6). The same pattern was recorded when the comparisons were based on the years 2018 and 2017 for different diameters: the differences among the datasets were significant when the analysis were done using all species (*F* = 2.61; *P* = 0.001) or common species (*F* = 2.75; *P* = 0.001), but not when the analysis was based on rare species data (*F* = 0.84; *P* = 0.77; Fig. S2).

**Table 2:** Results from generalized discriminant analysis based on distances testing differences between the data obtained in 2018 and the citizen science data gathered in different years (from 2010 to 2017). For each set of results, F-values were considered significant at *P* < 0.05/8 according to the Bonferroni method. df = residual degrees of freedom. Significant F- values are in bold.

	Year	df	F	P
Whole	2010	26	**5.51**	0.001
	2011	33	**4.42**	0.001
	2012	28	**3.13**	0.001
	2013	41	**3.57**	0.001
	2014	28	**4.16**	0.001
	2015	37	**6.54**	0.001
	2016	37	**5.47**	0.001
	2017	29	**4.15**	0.001
Common	2010	22	**4.17**	0.001
	2011	29	**4.00**	0.001
	2012	27	**2.86**	0.003
	2013	38	**3.13**	0.001
	2014	26	**4.02**	0.001
	2015	29	**5.50**	0.001
	2016	33	**5.05**	0.001
	2017	26	**3.81**	0.001
Rare	2010	14	2.54	0.019
	2011	22	0.95	0.489
	2012	19	1.21	0.303
	2013	24	1.89	0.034
	2014	16	0.82	0.565
	2015	22	2.32	0.020
	2016	18	2.43	0.019
	2017	17	1.03	0.443

## Discussion

Colonization of new man-made habitats is an important process that may counteract biodiversity loss^30^. Stormwater ponds is one category of such new man-made habitats and, therefore, it is important to examine whether knowledge on the biodiversity in the area surrounding the ponds based on citizen science data (CSD) can be used to predict the biodiversity in these ponds. Our results suggest that the diversity of rare species in these new man-made habitats could be predicted from the CSD. However, the results also suggest that CSD cannot be used to portray the overall biodiversity of dragonflies in stormwater ponds. There could be several reasons underlying this result.

First, there is a tendency that opportunistic CSD are biased towards over-reporting rare species and under-reporting more common species^20,21^. We found support for this bias because ordination scores obtained with our dataset and with CSD overlapped mainly when rare species were used in the analyses. Similarly, Snäll *et al*.^20^ found that common bird species were not regularly reported in the Swedish Species Observation System, and we suggest that the same holds for dragonflies. For example, *L. quadrimaculata* and *S. vulgatum* were found in 94.4% of the ponds in 2018, but in the CSD set (radius 10 km) these two species were reported, in average, with much lower frequencies (8.8 % and 21.6 %, respectively). In contrast, species less abundant, in our dataset of stormwater ponds, were reported with a similar frequency in the CSD-set. Currently, methods are being developed to correct for these biases^31^, and they should be used in future studies when predicting colonization of new man-made habitats. However, it is worth noting that citizen scientists may be especially interested in finding rare species with conservation interest^32^. Thus, we also found some support for this view, which suggests that even biased data may be beneficial for conservation purposes^24^.

Second, the stormwater ponds may not be comparable to the more natural habitats sampled in the CSD. For example, since the stormwater ponds are more recent habitats, they might be at early successional stages preferred by only certain dragonfly species. In contrast, CSD habitats might represent freshwater systems with a wider range of habitat characteristics or at later successional stages, which are preferred by a different set of dragonfly species. However, studies have found that aquatic insect communities in newly-created ponds may reach community structure similar to that in the natural ponds within one or two years^33–35^. Thus, since the youngest stormwater ponds were over four years old, we suggest that differences in successional stages were not the reason why we could not predict the biodiversity of stormwater ponds based on the CSD. In addition, studies using systematic protocols that were designed to compare the biodiversity of aquatic insects (including dragonflies) in stormwater ponds and more natural ponds have found similar results^11^.

Third, the CSD-set was collected from all kinds of aquatic habitats while the stormwater ponds probably represent a more similar range of aquatic habitats. If we had restricted our CSD-set to stormwater ponds, we might have found that CSD could predict the community composition in the stormwater ponds. Unfortunately, the CSD did not have enough replicates of stormwater ponds for such a comparison. Hence, we emphasize that our goal was to ask whether CSD collected from aquatic habitats could predict the community of Odonata in stormwater ponds.

We expected that the use of CSD collected from a larger diameter around our study area would make a better prediction, since a large area covers more habitats compared with more limited areas^36,37^. Conversely, one could expect a decrease in predictability with area, due to the distance decay of similarity in ecological communities^19,38^. However, we did not find support for any of these expectations because, independently of the diameter by which we based our comparison on, there was a significant difference in species composition between our stormwater pond data and the CSD. Thus, we did not observe that Odonata communities from aquatic habitats surveyed by volunteers, which were geographically closer to our study area, were more similar to the stormwater pond data. This finding suggests that under-reporting common species and over-reporting rare species still overrides the effect of area increase in our dataset.

We also found that variation in community structure (i.e., beta diversity) was significantly higher in the CSD compared to our stormwater pond data. We suggest that the main reason for this pattern is that stormwater ponds are more similar to each other than the water bodies from the CSD-set. The water bodies in the CSD included all kinds of freshwaters, from lentic to lotic and from temporary to large permanent lakes. Differences in biodiversity among different landscape or habitat types have also been found in previous studies on freshwater invertebrates. For example, urban and rural ponds may support different invertebrate communities (e.g.^39^), forest lakes and bog lakes typically harbor distinct invertebrate communities (e.g.^40^), and lotic and lentic ecosystems generally show contrasting invertebrate communities in terms of alpha and beta diversity (e.g.^41^). In this sense, our CSD-set should show overall higher levels of biodiversity than our stormwater pond dataset. An alternative, but not mutually exclusive, explanation for the difference in beta diversity could be the under-representation of common species in the CSD, which thereby could inflate the estimates of beta-diversity in the CSD.

In summary, we were unable to predict patterns in dragonfly biodiversity in stormwater ponds based on data collected by citizen scientists. We suggest that the main reason for this result is that common species are under-reported and rare species are over-reported by citizen scientists. Similar problems with the under-reporting of common species have been found in studies estimating annual variation in birds, species richness in beetles, and spatio-temporal variation in beetle abundance^9,19,20,42^. There is thus a need for predictive models that take into account or correct for the under-reporting of common species in CSD, and such models should provide better predictions of population trends and colonization of man-made habitats by species.

## Supplementary information

Supplementary information.

## References

[1] Venter, O. *et al*. Sixteen years of change in the global terrestrial human footprint and implications for biodiversity conservation. *Nature Communications* **7**, 10.1038/ncomms12558. (2016). 10.1038/ncomms12558 PMC4996975 27552116

[2] Troudet J (2017). Taxonomic bias in biodiversity data and societal preferences. Scientific Reports.

[3] Dickinson JL, Zuckerberg B, Bonter DN (2010). Citizen science as an ecological research tool: challenges and benefits. Annual Review of Ecology, Evolution, and Systematics.

[4] Dickinson JL (2012). The current state of citizen science as a tool for ecological research and public engagement. Frontiers in Ecology and the Environment.

[5] Silvertown J (2009). Trends in Ecology and Evolution.

[6] Devictor V (2010). Citizen science programmes as useful tools for conservation biogeography. Diversity and Distribution.

[7] Mair L (2016). Evaluating citizen science data for forecasting species responses to national forest management. Ecology and Evolution..

[8] Aceves‐Bueno (2017). The Accuracy of Citizen Science Data: A Quantitative Review. Bulletin of the Ecological Society of America.

[9] Snäll T (2014). Evaluating temporal variation in citizen science data against temporal variation in the environment. Ecography.

[10] Scher O, Thiery A (2005). Odonata, Amphibia and environmental characteristics in motorway stormwater retention ponds (Southern France). Hydrobiologia.

[11] Le Viol I, Mocq J, Julliard R, Kerbiriou C (2009). The contribution of motorway stormwater retention ponds to the biodiversity of aquatic macroinvertebrates. Biological Conservation.

[12] Hassall C, Anderson S (2015). Stormwater ponds can contain comparable biodiversity to unmanaged wetlands in urban areas. Hydrobiologia.

[13] Holtmann L (2018). Stormwater ponds promote dragonfly (Odonata) species richness and density in urban areas. Ecological Engineering.

[14] CEDR. Management of contaminated runoff water: current practice and future research needs. SBN: 979-10-93321-18-9 (2016).

[15] European Commission, The EU Floods Directive. [online] Available at, http://ec.europa.eu/environment/water/flood_risk/, [Accessed 16 March 2019] (2012).

[16] Corbet, P. S. Dragonflies: Behaviour and Ecology of Odonata. — Harley Books, Martins. (1999).

[17] Oertli B (2002). Does size matter? The relationship between pond area and biodiversity. Biological Conservation.

[18] Hassall C, Hollingshead J, Hull A (2011). Environmental correlates of plant and invertebrate species richness in ponds. Biodiversity and Conservation.

[19] Nekola JC, White PS (1999). The distance decay of similarity in biogeography and ecology. Journal of Biogeography.

[20] Snäll T (2011). Evaluating citizen-based presence data for bird monitoring. Biological Conservation.

[21] Gardiner MM (2012). Lessons from lady beetles: accuracy of monitoring data from US and UK citizen‐science programs. Frontiers in Ecology and the Environment.

[22] Johansson F (2019). Environmental variables drive differences in the beta diversity of dragonfly assemblages among urban stormwater ponds. Ecological Indicators.

[23] Sahlén, G. Sveriges Trollsländor. Fältbiologerna, Stochholm (1996).

[24] Tiago P, Pereira HM, Capinha C (2017). Using citizen science data to estimate climatic niches and species distributions. Basic and Applied Ecology.

[25] Anderson MJ, Willis TJ (2003). Canonical analysis of principal coordinates: a useful method of constrained ordination for ecology. Ecology.

[26] Anderson MJ, Robinson J (2003). Generalized discriminant analysis based on distances. Australian & New Zealand Journal of Statistics.

[27] Anderson MJ (2006). Distance‐based tests for homogeneity of multivariate dispersions. Biometrics.

[28] Anderson MJ, Ellingsen KE, McArdle BH (2006). Multivariate dispersion as a measure of beta diversity. Ecology Letter.

[29] Oksanen J (2017). Vegan: Community Ecology Package. R package version.

[30] Clobert, J., Baguette, M., Benton, T.G., & Bullock, J.M. Dispersal ecology and evolution. Oxford: Oxford Univ. Press. (2012).

[31] Bird TJ (2014). Statistical solutions for error and bias in global citizen science datasets. Biological Conservation.

[32] Losey JE, Perlman JE, Hoebeke ER (2007). Citizen scientist rediscovers rare nine-spotted lady beetle, *Coccinella novemnotata*, in eastern North America. Journal of Insect Conservation.

[33] Street M, Titmus G (1979). The colonisation of experimental ponds by Chironomidae Diptera. Aquatic Insects.

[34] Gee JHR, Smith BD, Lee KM, Griffiths SW (1997). The ecological basis of freshwater pond management for biodiversity. Aquatic Conservation: Marine and Freshwater Ecosystems.

[35] Bloechl (2010). Abundance, diversity and succession of aquatic Coleoptera and Heteroptera in a cluster of artificial ponds in the North German Lowlands. Limnologica.

[36] MacArthur R. H., & Wilson E. O. The theory of island biogeography. Princeton, NJ: Princeton University Press (1967).

[37] Kohn DD, Walsh DM (1994). Plant species richness – the effect of island and habitat diversity. Journal of Ecology.

[38] Soininen J, McDonald R, Hillebrand H (2007). The distance decay of similarity in ecological communities. Ecography.

[39] Hill MJ, Heino J, Thornhill I, Ryves DB, Wood PJ (2017). Effects of dispersal mode on the environmental and spatial correlates of nestedness and species turnover in pond communities. Oikos.

[40] Heino J (2013). Does dispersal ability affect the relative importance of environmental control and spatial structuring of littoral macroinvertebrate communities?. Oecologia.

[41] Williams P (2004). Comparative biodiversity of rivers, streams, ditches and ponds in an agricultural landscape in Southern England. Biological Conservation.

[42] Jeppson T (2010). The use of historical collections to estimate population trends: A case study using Swedish longhorn beetles (Coleoptera: Cerambycidae). Biological Conservation.

[43] *Lantmäteriet*, Geodataportalen. URL, https://www.geodata.se (2020).

