# Supplementary material for: Can information from citizen science data be used to predict biodiversity in stormwater ponds?
Source: Sci Rep. 2020 Jun 10;10:9380. doi: 10.1038/s41598-020-66306-0 (PMC7287044; doi:10.1038/s41598-020-66306-0)
Supplement: Supplementary file 1 — Supplementary information. [file 41598_2020_66306_MOESM1_ESM.docx]

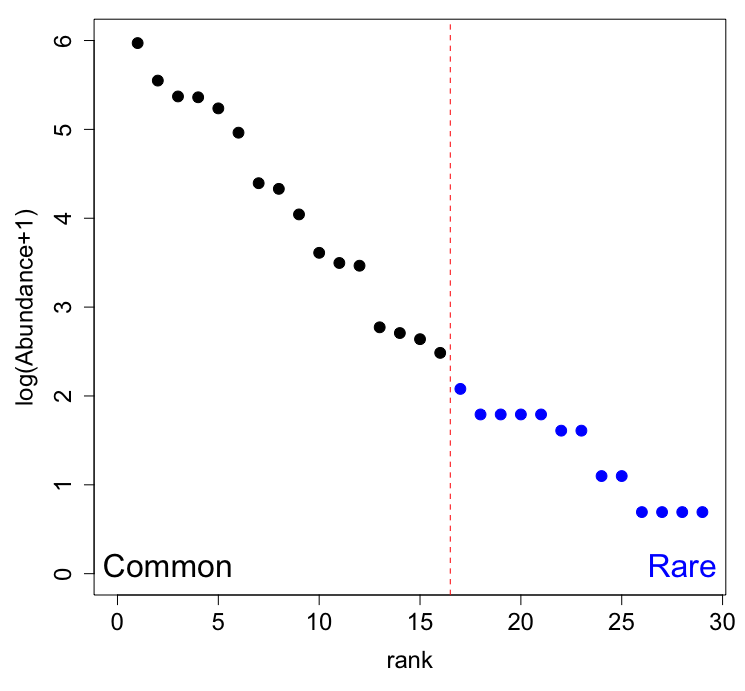


Figure S1. A Whittaker plot of dragonfly species in stormwater ponds. Shown is the log-transformed abundance from the most abundant to the least abundant species. The dashed line indicates the rank used to classify the species into common (black circles) and rare (blue circles).


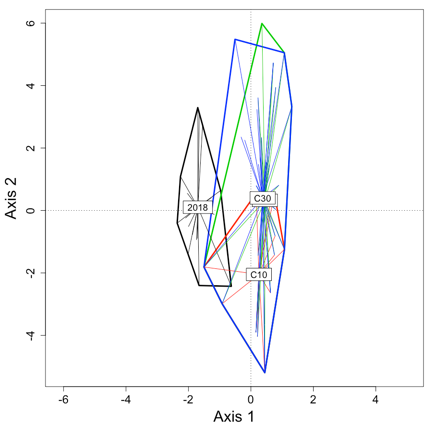

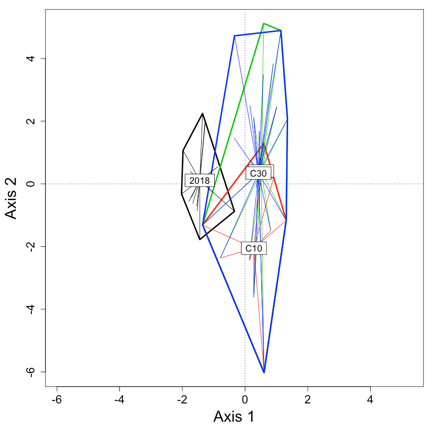

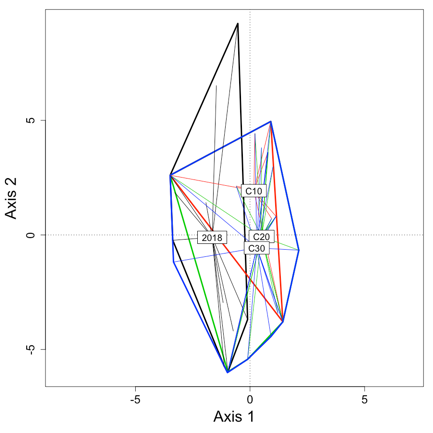


Figure S2. Ordination of sites (Canonical Analysis of Principal Coordinates) based on dragonfly species recorded in stormwater ponds (2018) and by citizen scientists (considering environments within diameters of 10 km, 20 km and 30 km around the stormwater ponds: C10, C20 and C30; respectively). Shown are the results obtained with all species (left), common species (center) and rare species (right) for the year 2017. Note that the labels for diameters 20 and 30 overlap in the figures for all and common species.
